# A Functional Polymorphism (rs10817938) in the XPA Promoter Region Is Associated with Poor Prognosis of Oral Squamous Cell Carcinoma in a Chinese Han Population

**DOI:** 10.1371/journal.pone.0160801

**Published:** 2016-09-13

**Authors:** Chunhai Gao, Jinzhu Wang, Chong Li, Wei Zhang, Guoxia Liu

**Affiliations:** 1 Department of Clinical Laboratory, Linyi People’s Hospital, Linyi, Shandong, P.R.China; 2 Jinan Stomatological Hospital, Jinan, Shandong, P.R.China; 3 Department of Rehabilitation, Linyi People’s Hospital, Linyi, Shandong, P.R.China; Duke Cancer Institute, UNITED STATES

## Abstract

Single nucleotide polymorphisms of *XPA* gene have been studied in several cancers such as rs10817938, rs2808668. However, the role of XPA polymorphisms in patients with oral squamous cell carcinoma (OSCC) remains unclear. Thus, we analyzed the association of XPA polymorphisms with OSCC risk, clinicopathological characteristics and prognosis in the present study. TaqMan genotyping was used to evaluate the frequency of rs10817938, rs2808668 polymorphisms in OSCC patients. The prognostic significance of these polymorphisms was evaluated using Kaplan-Meier curves, Log-Rank analyses, and the Cox proportional hazard model. Luciferase reporter assay, RT-PCR and western blot were used to determine whether rs10817938 could influence transcription activity and XPA expression. The results showed that individuals carrying TC and CC genotypes had significantly greater risk of developing OSCC (OR = 1.42, 95% CI 1.04–1.93; OR = 2.75, 95% CI 1.32–5.71, respectively) when compared with wild-type TT genotype at rs10817938. OSCC patients with C allele at rs10817938 were more susceptible to lymph metastases, poor pathological differentiation and late TNM stage (OR = 1.67, 95% CI 1.17–2.37; OR = 1.64, 95% CI 1.18–2.28; OR = 1.54, 95% CI 1.11–2.14; respectively). A significant gene-environment interaction between smoking and CC genotype at rs10817938 was observed (COR = 3.60, 95% CI 1.20–10.9) and data also showed that OSCC patients with CC genotype and C allele had worse survival (*p<*0.001 for both). The T to C substitution at rs10817938 significantly decreased transcription activity of *XPA* gene, XPA mRNA and protein were also decreased in individuals with C allele at rs10817938. In addition, no significant association of rs2808668 polymorphism with OSCC risk, prognosis could be observed. In conclusion, the present study showed that XPA rs10817938 polymorphism is a functional SNP in *vitro* and in *vivo* and a biomarker for poor prognosis in OSCC patients.

## Introduction

Oral squamous cell carcinoma (OSCC), one of the most lethal malignancies, remains the most common oral cavity cancer, accounting for >274,000 newly diagnosed cancer cases annually worldwide[1]. Due to late detection and later stages when diagnosed, OSCC is frequently accompanied by metastasis, high recurrence and poor prognosis[2]. Despite progress in early detection and treatment, mortality is largely unchanged and the 5-year survival rate worldwide for OSCC remains ~50%[3]. Thus, improvements in current knowledge of molecular changes associated with OSCC are urgently needed for exploring novel methods of diagnosis and prognosis. The combination of the susceptible genetic background and environmental factors is identified to contribute to the development of OSCC. Although smoking and drinking are well-established risk factors, OSCC could develop in the absence of tobacco and alcohol use[4]. Thus, genetic susceptibility to OSCC should be further investigated and paid more attention. For instance, an impaired ability to repair DAN damaged by mutagens was identified to play a important role in OSCC pathogenesis[4].

DNA repair system plays a vital role in maintaining the integrity and stability of the genome and defending mutations, which chiefly includes nucleotide excision repair (NER), base excision repair (BER), mismatch repair (MMR) and double-strand break repair (DSBR)[5]. The NER pathway is an important cellular defense against diverse structurally unrelated DNA lesions, which can monitor and repair DNA damage caused by endogenous and exogenous factors[6]. NER processes include several stages including damage recognition, demarcation, unwinding, incision, as well as new strand ligation, all of which require corresponding functional proteins[7,8]. The *xeroderma pigmentosum A* (*XPA*) gene product is a zinc-finger DNA-binding protein involved in damage recognition with an affinity for damage that targets the core of NER system. XPA is the first human NER protein with a preference for binding to damaged DNA and it maintains an intricate network of contacts with core repair factors during DNA repair process[9].

Previous studies demonstrated that polymorphisms in DNA damage response genes could increase the risk of development cancer[10–12]. Polymorphisms of core NER genes such as *XPA*, *XPC*, and *ERCC* can change NER ability by influencing expression and function of important proteins, thereby altering individual susceptibility to cancer[13,14]. Concerning XPA polymorphism, the most widely studied polymorphism is rs1800975 (-4G>A), or the XPA (-4) G to A substitution, which is located in the 5’-untranslated region (UTR) and is four nucleotides upstream of the start codon[15]. To date, numerous molecular epidemiological studies have been conducted to elucidate the association between rs1800975 and different types of cancer[14,16–18]. In addition to rs1800975, recent studies have indicated a potential association of a novel promoter polymorphism (rs10817938) in the *XPA* loci and cancer development[19]. rs10817938 polymorphism is a T to C substitution located -2718 bp from the transcriptional start site within *XPA* gene. Previous studies have explored an association of rs10817938 polymorphism with hepatocellular cancer risk [19]. rs2808668 is also a T to C substitution located -514bp from the transcriptional start site. Previous studies have found the positive association between rs2808668 polymorphism and lung cancer, breast cancer risk[17,20]. However, no report has been provided to show the association of these two polymorphisms with OSCC. Thus, we studied the distribution of rs10817938 and rs2808668 in OSCC patients, and assessed the association of these polymorphisms with clinicopathological characteristics and prognosis. Furthermore, we conducted molecular studies to confirm whether rs10817938 polymorphism had the potential to influence *XPA* expression.

## Materials and Methods

### Study Subjects

A total of 362 newly diagnosed and histologically confirmed OSCC patients were enrolled in the present study from September 2010 to October 2012 from the Linyi’s People Hospital (Shandong, China) and the Jinan Stomatological Hospital (Shandong, China). As controls, 350 gender- and ethnicity-matched, unrelated healthy individuals were included. All controls received the physical examination and none had a history of cancer during the same periods in the same hospitals as the OSCC patients. All aspects of the present study were approved by the Research Ethics Committee of Linyi’s People Hospital (Ethical Approval Number: SDLYYY201006078, Linyi, China) and the Jinan Stomatological Hospital (Ethical Approval Number: JNKQYY20100400108, Jinan, China), and all procedures were carried according to the principles of the Declaration of Helsinki. Written informed consent was obtained from all participants.

### Medical data extraction and follow-up

Clinical and laboratory information from OSCC patients were extracted from medical records at the time of diagnosis including age, gender, smoking, drinking, tumor location, lymph node metastasis, TNM stage, tumor differentiation. OSCC patients were recruited for the follow-up period after surgery. The final follow-up was on October 22, 2015, with follow-up times ranging from 2 to 58 months.

### XPA polymorphisms genotyping

TaqMan SNP genotyping assays were used to confirm genotypes for rs10817938 and rs2808668 at the *XPA* gene. Approximately 2 ml blood was collected from each subject into an EDTA tube. Genomic DNA was isolated using a Genomic DNA kit (Axygen, CA, USA) according to the manufacturer’s instructions and stored at -20°C until use. Genotyping of rs10817938 and rs2808668 polymorphisms was performed using custom TaqMan SNP genotyping assays (C__31012492_20 for rs10817938 and C__9312100_20 for rs2808668, ThermoFisher, OK, USA). Genotyping and allele analysis were conducted with TaqMan genotyping master mix (ThermoFisher) and an ABI Prism 7900HT genetic detection system according to the manufacturer’s instructions in a final volume of 25 μL including 12.50 μL master mix, 1.25 μL of assay Mix, 11.25 μL of ddH20. PCR conditions were as follows: 95°C for 15 s, 60°C for 1 min, for 40 cycles. Genotype and allelic frequency were calculated based on allelic discrimination plots using automatic allele analysis. Genotype reproducibility was evaluated in 10% duplicate samples and data were 100% concordant.

### Construction of XPA promoter-reporter plasmids

rs10817938 polymorphism was located in the promoter of the *XPA* gene so we used a luciferase reporter assay to study any effect on *XPA* gene expression *in vitro*. First, we created XPA promoter-luciferase reporter plasmids containing either rs10817938 with T or C allele by amplifying the 300-bp promoter region (from -2519 to -2818) using PCR. DNA templates isolated from patient blood samples. Primers were 5’-CGGGGTACCATGCTAGTACTGCTACCAG-3’ and 5’-CCGCTCGAGATTCAGTAGCTGTCCTGAC-3’. Cleavage sites of *KpnI* and *XhoI* were also introduced at the 5’-end of forward and reverse primers, respectively. To confirm the matched nucleotides and the plasmid containing either the rs10817938 T or C allele, amplified fragments were sequenced. Both amplified fragments and pGL3-basic vector (Promega, MD, USA) were digested with *KpnI* and *XhoI* enzymes (NEB, BioLabs Inc., CA, USA). After digestion, amplified fragments were cloned into pGL3-basic reporter vector (Promega, MD, USA). After cloning and amplification, the vectors were sequenced again to confirm no orientation errors and integrity of each construct.

### Cell culture

Human OSCC cell lines SCC-4(ATCC^®^CRL-1624™), SCC-9(ATCC^®^CRL-1629^TM^), SCC-25(ATCC^®^CRL-1628^TM^) and HeLa cells (Purchased from the Institute of Cellular Research, Chinese Academy of Science, Shanghai, China; No. TCHul87) were cultured in DMEM medium containing 10% fetal bovine serum (Gibco, CA, USA), 100 units/mL penicillin, and 100 μg/mL streptomycin (Gibco, CA, USA). Cells were incubated in a humidified atmosphere of 5% CO_2_ at 37°C.

### Transfection and luciferase assay

The pGL3-basic vector without an insert was used as a negative control. As an internal control, *Renilla* luciferase (pRL-SV40 vector) was used to normalize and reduce differences in transfection efficiencies and subsequent variations in experiments. Before transfections, OSCC and HeLa cells were seeded in 24-well plates and co-transfected with recombinant pGL3 luciferase reporter containing the rs10817938 T or C allele, the pGL3-basic vector or the pRL-SV40 vector using PolyJet DNA *in vitro* transfection reagent (Signagen Laboratories, MD, USA) according to the manufacturer’s instructions. Luciferase assay was performed with a Dual Glo luciferase system (Promega, MD, USA). After 24 h incubation, cells were lysed in passive lysis buffer. Luciferase signals were measured with a dual-luciferase reporter assay system and a FLx800™ multi-detection microplate reader (BioTek, Winooski, USA). All luciferase activity was normalized against *Renilla* luciferase gene activity and measurements of fluorescent intensity were expressed as means ± SE of firefly/*Renilla* obtained from three readings at each setting. Independent triplicate wells were used for each vector.

### RNA isolation and quantitative real-time PCR

Total RNA was isolated from all 362 OSCC tumor tissue using TriZol reagent (Invitrogen, CA, USA) according to the manufacturer’s instructions. An aliquot of total RNA (1 μg) from each sample was used for cDNA synthesis with a reverse transcription kit (Takara, Japan). Reverse transcription was conducted at 35°C for 10 min, followed by 95°C for 5 s. After obtaining cDNA, quantitative real-time PCR (qRT-PCR) was performed (20 ul final volume), with qRT-PCR conditions as follows: 98°C for 10 s, 60°C for 15 s, 72°C for 30 s, for 40 cycles. XPA mRNA expression was estimated relative to GAPDH using the equation 2^-ΔΔCt^(ΔCt = Ct_XPA_-Ct_GAPDH_). Primers were: 5′- GACACAGGAGGAGGCTTCAT -3′ (forward) and 5′-TGCAGTTATCACAAGTTGGCA -3′ (reverse) for XPA. And 5′- CCAGAACATCATCCCTGCCT-3′ (forward) and 5′- CCTGCTTCACCACCTTCTTG-3′ (reverse) for GAPDH.

### Protein isolation and Western blot

According to the distribution of rs10817938 genotypes, all 362 OSCC patients were divided into three groups. To analyze the correlation between rs10817938 genotypes and protein expression *in vivo*, we randomly selected 26 OSCC tumor tissues and isolated protein. Frozen tumor tissues were homogenized on ice and lysed in RIPA buffer containing protease inhibitor (Complete, Sigma, CA, USA) and phenylmethanesulfonyl fluoride (PMSF). Homogenates were sonicated and centrifuged at 12,000 rpm at 4°C for 5 min to remove cell debris. Protein was measured using the BCA assay kit (Beoytime Biotech, Shanghai, China). Then, ~30 μg of total protein was separated via 8% SDS-PAGE and transferred to polyvinylidene difluoride membranes (Millipore, MA, USA). Membranes were blocked with 5% non-fat milk in TBS buffer containing 0.1% Tween-20, and then incubated with human XPA antibody (1:1,000, sc-28353, mouse monoclonal IgG, Santa Cruz) and GAPDH antibody (1:1,000, sc-32233; mouse monoclonal IgG, Santa Cruz) at 4°C overnight. Horseradish peroxidase (HRP)-conjugated anti-mouse IgG was used as secondary antibody (1:5,000, Beoytime Biotech, Shanghai, China). Signals were captured using the CCD camera system (Bio-Rad) with the HRP chemiluminescent kit (Beoytime Biotech, Shanghai, China). XPA protein expression was normalized to GAPDH.

### Statistical analysis

Continuous data were compared between OSCC patients and controls using the Student’s t-test, and categorical data were compared using a Chi-square test. The association between genotypes and OSCC risk was estimated with odds ratios (ORs) using an unconditional logistic regression model. A gene-environment interaction between XPA polymorphisms and smoking, or drinking was via a case-case design and estimated case-only odds ratios (CORs) [21]. Survival probabilities were estimated with Kaplan–Meier analysis, and significant differences were analyzed with the log-rank test. Cox proportional hazards models were used to analyze associations between XPA polymorphisms with OSCC survival. Hazard ratios (HR) and 95% confidence intervals (CI) were estimated using multivariable models. Deviations from Hardy-Weinberg equilibrium were assessed with a Chi-squared test. Difference in XPA expression among genotypes was also compared with a Student’s t-test.

The false-positive report probability (FPRP) was calculated to assess the significant findings[22,23]. 0.2 was set as an FPRP threshold and assigned a prior probability of 0.1 to detect an odds ratio (OR) of 0.67/1.50 (protective/risk effects) for the association with rs10817938 genotypes, alleles. Only the significant result with an FPRP value less than 0.2 was considered a noteworthy finding.

All statistical analyses were performed with SPSS 22.0 (SPSS Inc., Chicago, IL) and GraphPad Prism 6.0 (GraphPad, CA, USA). *p<*0.05 was considered statistically significant.

## Results

### Participants’ characteristics

OSCC patients (N = 362) and non-carrier controls (N = 350) were studied and all participants were of Han ethnicity from the same region in Shandong province of China. There were no statistical differences in age, gender, smoking and drinking status between OSCC patients and normal controls (*p>*0.05 for all, Table 1).

**Table 1:** Clinical characteristics of OSCC patients and controls.

Characteristics	OSCC patients (n = 362)	Normal controls (n = 350)	P value
Age (Year)	55.1 ±9.7	53.8 ±10.2	0.11
Gender (n, %)			
Male	235 (64.9)	238 (68.0)	0.37
Female	127 (35.1)	112 (32.0)	0.29
Tobacco Smoking			
Smoker	252 (69.6)	226 (64.6)	0.28
Non-Smoker	110 (30.4)	124 (35.4)	0.17
Tobacco Smoking(Pack-year)			
<20	92	101	024
20–40	89	78	0.31
>40	71	47	0.07
Alcohol Drinking			
Drinker	207 (57.2)	183 (52.3)	0.25
Non-Drinker	155 (45.6)	167 (47.7)	0.23
Smoking and Drinking			
Smoking or/and Drinking	340	325	0.48
Never smoking and drinking	22	25	0.35
Tumor Location			
Tongue	140		
Gingiva	127		
Other	95		
TNM Stage			
I/II	193 (53.3)		
III/IV	169 (46.7)		
Lymph Node Metastasis			
Negative	204 (56.4)		
Positive	158 (43.6)		
Distant Metastasis			
Negative	356 (98.3)		
Positive	6 (1.7)		
Tumor Differentiation			
Well	138 (38.1)		
Moderate	126 (34.8)		
Poor	98 (27.1)		

### XPA polymorphisms and OSCC risk

Distribution and statistical analyses of rs10817938 and rs2808668 genotypes in OSCC patients and normal controls were summarized in Table 2. The values for the χ2 tests of HWE were 0.191 and 0.112 for rs10817938 and rs2808668 in normal controls, respectively. Significant differences in the distribution of rs10817938 were found between OSCC patients and normal controls. Compared with the wild-type genotype (TT) at rs10817938, individuals with TC and CC genotypes had significantly increased risk for OSCC (OR = 1.42, 95%CI 1.04–1.93; OR = 2.75, 95%CI 1.32–5.71, respectively). Significantly increased risk for OSCC development was also noted for those with the C allele at rs10817938 compared with the T allele (OR = 1.48, 95%CI 1.16–1.89). However, we did not observe any positive association between rs2808668 polymorphism and OSCC risk. Although the frequencies of CC genotype and C allele were a little bit higher in OSCC patients, there was no statistically significant difference (OR = 1.11, 95%CI 0.74–1.67; OR = 1.05, 95%CI 0.85–1.30; respectively).

**Table 2:** Genotype distribution and allele frequencies for rs10817938 at *XPA* gene in OSCC patients and normal controls.

	OSCC Patients (n, %)	Controls (n, %)	HWE	OR	95% CI	*P* value
rs10817938			0.191			
Genotype T/T	186 (51.4)	216 (61.7)		1	-	
T/C	150 (41.4)	123 (35.1)		1.42	1.04–1.93	0.03
C/C	26 (7.2)	11 (3.2)		2.75	1.32–5.71	<0.01
T/C+C/C	176 (48.6)	134 (38.3)		1.53	1.13–2.06	<0.01
Allele						
T	522 (72.1)	555 (79.3)		1	-	
C	202 (27.9)	145 (20.7)		1.48	1.16–1.89	<0.01
rs2808668			0.112			
Genotype T/T	122 (33.7)	120 (34.3)		1	-	
T/C	161 (44.5)	160 (45.7)		0.99	0.71–1.38	0.51
C/C	79 (21.8)	70 (20.0)		1.11	0.74–1.67	0.35
T/C+C/C	240 (66.3)	230 (65.7)		1.03	0.75–1.340	0.47
Allele						
T	405 (55.9)	400 (57.1)		1	-	
C	319 (44.1)	300 (42.9)		1.05	0.85–1.30	0.34

Adjusted for age, gender, smoking and drinking status.

### Association between XPA polymorphisms and clinicopathological features in OSCC patients

The association of these two polymorphisms with clinicopathological features of OSCC patients was evaluated and stratification analysis by age, gender, tobacco and alcohol use, tumor location, TNM stages, lymph metastasis and tumor differentiation were further performed (Table 3). Positive associations between rs10817938 polymorphism and metastasis, TNM stage, and tumor differentiations were noted (OR = 2.76, 95%CI 1.17–6.51; OR = 2.78, 95%CI 1.19–6.46); OR = 4.54, 95%CI 1.94–10.6; respectively) and all 6 OSCC patients with distant metastasis were of CC genotype at rs10817938. Similarly, TC genotype at rs10817938 was significantly associated with lymph metastasis (OR = 1.69, 95%CI 1.10–2.62). At the allele level, OSCC patients carrying a C allele at rs10817938 had greater risk for lymph metastasis, late TNM stage and poor tumor differentiation (OR = 1.67, 95%CI 1.17–2.37; OR = 1.64, 95%CI 1.18–2.28; OR = 1.54, 95%CI 1.11–2.14; respectively). The gene-environment assessment revealed a significant association between smoking and CC genotype: smokers with CC have ~260% increased risk for OSCC than non-smokers carrying the TT genotype (COR = 3.60, 95% CI 1.20–10.9). Similar data were obtained regarding smokers with the C allele (COR = 2.13, 95% CI 1.44–3.15). All 26 OSCC patients with CC genotype were smoker or/and drinker. In addition, no significant association between rs2808668 polymorphism with clinicopathological characteristics could be observed in OSCC patients.

**Table 3:** Association of rs10817938, rs2808668 polymorphisms with clinicopathological characteristics of OSCC patients.

Characteristics	rs10817938					rs2808668				
	Genotype			Allele		Genotype		Allele		
	T/T	T/C	C/C	T	C	T/T	T/C	C/C	T	C
Age										
<55	96	81	14	273	109	65	89	37	219	163
>55	90	69	12	249	93	57	72	42	186	156
OR (95% CI)	1.0	0.91 (0.59–1.40)	0.91 (0.40–2.08)	1.0	0.94 (0.67–1.30)	1.0	0.92 (0.58–1.48)	1.29 (0.73–2.28)	1.0	1.13 (0.84–1.51)
Gender										
Male	67	50	10	184	70	40	56	31	136	118
Female	119	100	16	338	132	82	105	48	269	201
OR (95%)	1.0	1.13 (0.72–1.77)	0.90 (0.39–2.10)	1.0	1.03 (0.73–1.44)	1.0	0.92 (0.56–1.51)	0.76 (0.42–1.36)	1.0	0.86 (0.63–1.17)
Tobacco Smoking										
Non-Smoker	74	33	3	180	40	38	51	21	127	93
Smoker	112	117	23	342	162	84	110	58	278	226
OR (95%)	1.0	1.49 (0.48–4.64)	**3.60 (1.20–10.9)**	1.0	**2.13 (1.44–3.15)**	1.0	0.98 (0.59–1.62)	1.25 (0.67–2.34)	1.0	1.11 (0.81–1.53)
Tobacco Smoking(Pack-year)										
<40	89	84	8	262	100	60	78	43	198	164
>40	23	33	15	79	63	24	32	15	80	62
OR (95%)	1.0	1.52 (0.83–2.80)	**7.26 (2.74–19.2)**	1.0	**2.09 (1.40–3.13)**	1.0	1.02 (0.55–1.92)	0.87 (0.41–1.86)	1.0	0.94 (0.63–1.38)
Alcohol Drinking										
Non-Drinker	85	57	13	227	83	46	73	36	155	145
Drinker	101	93	13	295	119	76	88	43	240	174
OR (95%)	1.0	1.37 (0.89–2.13)	0.84 (0.37–1.92)	1.0	1.10 (0.79–1.54)	1.0	0.73 (0.45–1.18)	0.72 (0.41–1.28)	1.0	0.78 (0.58–1.05)
Smoking and Drinking										
Never smoking and drinking	17	5	0	39	5	10	6	6	26	18
Smoking or/and Drinking	169	145	26	483	197	112	155	73	379	301
OR (95%)	1.0	**2.92 (1.05–8.10)**	**-**	1.0	**3.18 (1.24–8.19)**	1.0	2.31 (0.82–6.53)	1.09 (0.38–3.12)	1.0	1.15 (0.62–2.13)
Tumor Location										
Tongue/Gingiva	141	107	19	389	145	90	117	60	297	237
Other	45	43	**7**	133	**57**	32	44	19	108	82
OR (95%)	1.0	1.26 (0.77–2.05)	1.15 (0.46–2.92)	1.0	1.15 (0.80–1.66)	1.0	1.06 (0.62–1.80)	0.89 (0.46–1.71)	1.0	0.95 (0.68–1.33)
TNM Stage										
I/II	110	74	9	294	92	66	81	46	213	173
III/IV	76	76	17	228	110	56	80	33	192	146
OR (95%)	1.0	1.50 (0.97–2.31)	**2.76 (1.17–6.51)**	1.0	**1.5 (1.11–2.14)**	1.0	1.16 (0.73–1.87)	0.85 (0.48–1.50)	1.0	0.94 (0.70–1.26)
Lymph Metastasis										
Negative	118	76	10	312	96	70	94	40	234	174
Positive	68	74	16	210	106	52	67	39	171	145
OR (95%)	1.0	**1.69 (1.10–2.62)**	**2.78 (1.19–6.46)**	1.0	**1.64 (1.18–2.28)**	1.0	0.96 (0.60–1.55)	1.31 (0.74–2.32)	1.0	1.14 (0.85–1.53)
Tumor Differentiation										
Well + Moderate	143	110	11	396	132	87	120	57	294	234
Poor	43	40	15	126	70	35	41	22	111	85
OR (95%)	1.0	1.33 (0.81–2.20)	**4.54 (1.94–10.6)**	1.0	**1.67 (1.17–2.37)**	1.0	0.85 (0.50–1.44)	0.96 (0.51–1.80)	1.0	0.96 (0.69–1.34)

Gene-environment interaction was analyzed using a case–case design and estimating case-only odds ratios (CORs)

The FPRP values for significant findings at different prior probability levels are shown in S1 Table. Positive associations of rs10817938 allele with OSCC risk, smoking, TNM stage, lymph metastasis and tumor differentiation were found, because their probability to be a false-positive result was less than 20%. In contrast, we observed greater FPRP values for the significant associations between CC genotype and OSCC risk, clinicopathological characteristics when compared with TT genotype, suggesting some possible bias in these findings due to reduced sample size in these subgroups.

### Correlation analysis of XPA polymorphisms and prognosis in OSCC patients

**Fig 1:**
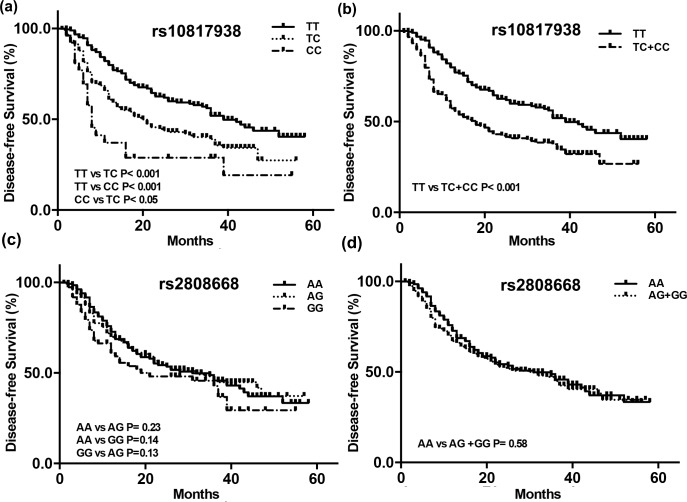
**Kaplan-Meir survival curves;** Kaplan-Meir survival curves for OSCC patients with rs10817938 (a, b) and rs2808668 (c, d) polymorphisms. The XPA rs10817938 polymorphism was correlated with the disease-free survival in OSCC patient, but not rs2808668.

Kaplan-Meier curves to evaluate the association between rs10817938, rs2808668 polymorphisms and survival indicated that survival differences occurred among OSCC patients with different rs10817938 genotypes, but not rs2808668 (Fig 1). Kaplan-Meier curves and log-rank tests confirmed that OSCC patients with CC genotype at rs10817938 had shorter survival than those with TC and TT genotypes (Fig 1A, *p<*0.001 for both). OSCC patients carrying C allele (TC+CC) at rs10817938 had worse survival compared with the wild-type TT genotype (Fig 1B, *p<*0.001). Multivariate Cox proportional hazards analysis data was presented in Table 4. Late TNM stage and tumor metastasis were two independent risk factors for poor survival of OSCC patients. Also, TC+CC carriers at rs10817938 had significantly greater risk of poor survival *versus* TT genotype (HR = 1.78, 95% CI 1.02–2.97), suggesting that the TC+CC genotypes at rs10817938 was an independent risk factor for poor survival.

**Table 4:** Multivariate Cox proportional hazard analysis of prognostic factors for OSCC patient OS.

Factors	Categories	Multivariate	
		HR (95% CI)	*p*
Age (Year)	>55 /<55	1.03 (0.66–1.92)	0.37
Gender	Male/Female	0.87 (0.57–2.04)	0.64
Tobacco Smoking	Yes/No	1.54 (0.79–2.76)	0.31
Alcohol Drinking	Yes/No	1.48 (0.68–2.44)	0.19
Tumor differentiation	Well+Moderate/Poor	2.43 (0.61–3.46)	0.36
Lymph Metastasis	Yes/No	3.17 (1.28–4.66)	<0.05
Distant Metastasis	Yes/No	4.02 (2.39–6.81)	<0.01
TNM Stage	III+IV/I+II	2.56 (1.77–4.29)	<0.01
Genotype at rs2808668	AG+GG/AA	1.24 (0.78–2.11)	0.21
Genotype at rs10817938	TC+CC/TT	1.78 (1.02–2.97)	<0.05

HR: Hazard Ratio; 95% CI: 95% Confidence Interval; m, months

### Effects of rs10817938 polymorphism on transcriptional activity

**Fig 2:**
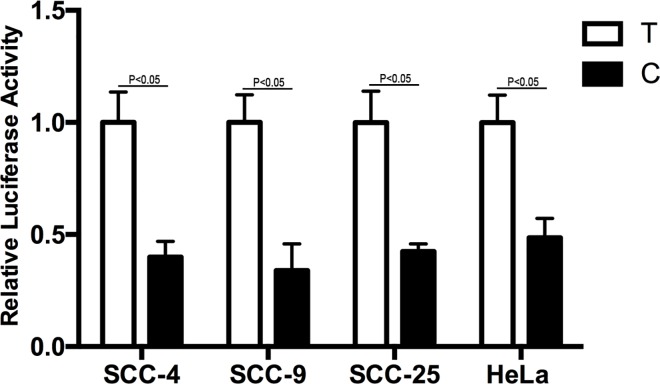
Effect of rs10817938 polymorphism on XPA promoter activity; Schematic representation of reporter plasmids containing a T or C allele at rs10817938, which was inserted upstream of the luciferase reporter gene in the pGL3 Basic plasmid. pRL-SV40 were co-transfected into SCC-4, SCC-9, SCC-25, HeLa cells as an internal control of *Renilla luciferase*. Columns are means from three independent experiments and bars are standard deviations.

To evaluate whether rs10817938 polymorphism could affect XPA promoter activity, we constructed luciferase reporter vectors (pGL3) with either a T or C allele and used them for transient transfections with SCC-4, SCC-9, SCC-25 and HeLa cells. Compared with the T allele, vectors with rs10817938 C allele had less relative luciferase activity in all four cell lines (*p<*0.05 for all, Fig 2). Thus, rs10817938 C allele could decrease transcriptional activity of the *XPA* gene *in vitro*.

### Association of rs10817938 polymorphism with mRNA and protein expression levels of XPA

**Fig 3:**
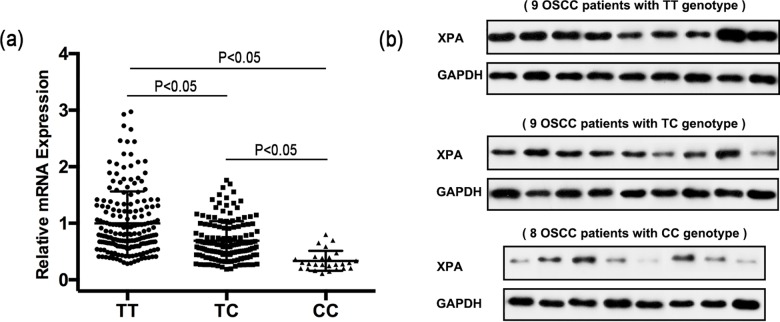
Association between rs10817938 polymorphism and XPA mRNA and protein expression in OSCC patients. (a) XPA mRNA expression in OSCC tumor tissues from individuals with different rs10817938 genotypes was measured using qRT-PCR; (b) XPA protein was measured with western blot in three groups of OSCC patients. OSCC patients with TT genotype (n = 9), TC genotype (n = 9) or CC genotype (n = 8).

Next, we assessed the effect of rs10817938 on XPA mRNA and protein expression *in vivo*. The results showed that XPA mRNA were significantly decreased in OSCC patients with TC and CC genotypes (*p<*0.05 for both, Fig 3A). Individuals with CC genotype also had relatively less XPA mRNA level than those with TC (*p<*0.05, Fig 3A). We then randomly selected 26 tumor samples from all OSCC patients and measured XPA protein expression level. In consistent with mRNA expression level, the results confirmed that OSCC patients with TC or CC genotype had relatively less XPA protein levels than those with TT genotype (Fig 3B, S1 Fig). Taken together, the observation of lower levels of *XPA* mRNA and protein expression in OSCC patients with CC genotype than those with other genotypes suggested that CC genotype at rs10817938 was a risk factor for OSCC.

## Discussion

NER is a multi-protein-mediated sequential pathway, and XPA is a critical protein for its initiation[24]. Previous studies demonstrated that polymorphisms in *XPA* genes could modulate NER efficiency and contribute to carcinogenesis [25]. In the present study, we found the significant association of a novel XPA functional polymorphism (rs10817938) and OSCC risk, poor prognosis. Allele C and genotype CC at rs10817938 could increase the risk of OSCC development and were associated with metastases, tumor pathological differentiation and TNM stages. AG+GG at rs10817938 was significantly associated with poor prognosis, and was identified as an independent risk factor for poor prognosis. In addition, we observed that the T to C substitution at rs10817938 could significantly decrease transcription activity of the *XPA* gene and XPA expression was down-regulated in individuals carrying the C allele. Taken together, the present study suggested that rs10817938 is functional polymorphism *in vitro* and *in vivo* and is a biomarker for risk and prognosis of OSCC.

XPA, as a zinc binding protein with an affinity for various types of DNA damage, was the first human NER protein documented to prefer to bind to damaged DNA and function at the core of the NER system [26]. XPA is located on chromosome 9q22.3, and rs10817938 polymorphism we investigated is located -2718 bp from the transcription start site. This polymorphism has been studied in patients with hepatocellular cancer, and rs10817938 CC genotype conferred relatively higher risk for HCC compared with the wild type TT genotype or TT+TC [19]. In agreement with these data, we noted that the CC genotype (or allele C) was a risk factor for OSCC and we also observed that this mutation was correlated with poor prognosis in OSCC patients. The positive association between XPA polymorphism and OSCC risk is biologically plausible because XPA is important in the NER pathway and the deficiency of XPA was reported to be related to OSCC susceptibility [27]. Identifying a risk XPA polymorphism is good for researchers to understand the genetic susceptible background of OSCC patients. As for clinical practice, XPA polymorphism could be used as important biomarker to screen those with high risks to OSCC in Chinese population.

In 2006, Sugimura reported the association between XPA rs1800975 polymorphism with OSCC risk in Japanese population[28]. Similar to other cancers, this group reported that AG and GG genotypes of rs1800975 polymorphism conferred significantly increased risk in OSCC patients relative to AA genotype. Wang reported that rs1800975 polymorphism was associated with increased risk of oral premalignant lesion (OPL) [29] and a gene-smoking interaction was noted between rs1800975 and smoking status in OPL patients, suggesting the rs1800975 mutation with smokers was associated with more OPL risk. Consistent with this phenomenon, our data show a gene-smoking interaction between the rs10817938 polymorphism and tobacco smoking: smokers with the CC genotype have greater risk for the development of OSCC. Tobacco contains several carcinogenic substrates such as polycyclic aromatic hydrocarbons which could cause DNA damage by covalent binding or oxidation [29,30,31]. NER is the major pathway for the repairs of tobacco carcinogen-induced bulky DNA adducts. Spitz’s group observed less DNA repair capacity in lung cancer patients than healthy controls, and this reduction could be modulated by tobacco [32]. Thus, as a tobacco-related malignancy, it is biologically plausible that polymorphisms of NER genes may predispose an individual to OSCC.

rs2808668 is another polymorphism located in *XPA* gene. It remains controversial on the association of rs2808668 with different type of cancers risk. In lung cancer and breast cancer, previous studies reported that rs2808668 polymorphism was significantly associated with cancer susceptibility[17,20]. However, no significant association between rs2808668 polymorphism with pancreatic cancer, larynx cancer could be observed[33,34]. Although rs2808668 showed a protective effect in atrophic gastritis, rs2808668 was not found to be significantly associated with the risk of gastric cancer[13]. Similarly, we did not find the positive association between rs2808668 polymorphism and OSCC risk, prognosis.

In tumors, low expression of XPA mRNA and proteins may cause defective DNA damage repair and be related with adverse tumor outcomes such as metastasis and poor pathological differentiation [35]. Here, we report that rs10817938 polymorphism was associated with low expression of *XPA* gene and this may impair the NER system and increase DNA damage. Molecular epidemiologic findings indicated that XPA rs10817938 polymorphism could increase the risk for OSCC, and this was consistent with functional analysis. The polymorphism’s effect on DNA repair capacity (DRC) of XPA has been documented to be associated with gene and protein expression, function, and stability [36,37]. For example, rs1800975 polymorphism at the 5‘-non-coding region of *XPA* gene causes suboptimal DRC that affects recognition and confirmation of DNA damage [38]. rs1800975 polymorphism was located at four nucleotides upstream of the ATG start codon (Kozak sequence), which may affect transcriptional and post-transcriptional gene expression [39,40]. The latter may occur via modulation of the mRNA tertiary structure and stability or binding between translational factors and mRNA [9]. In the present study, although we found rs10817938 polymorphism could affect transcriptional activity of *XPA* gene, the specific mechanism was not clear. One possibility we speculate may be that the T to C substitution of this polymorphism could affect the binding affinity of some transcriptional factor, which will be addressed in future functional studies.

Our study is limited by potential selection bias as a hospital-based case-control study. First, our sample size was relatively small, which may limit accuracy and reliability of results. Second, although we observed that rs10817938 polymorphism decreased transcriptional activity of the *XPA* gene, how this occurs was unclear. Next, the function or power of this polymorphism as a major predictive factor of OSCC development or prognosis in clinical practice was relatively low. Because this work was conducted in a Chinese Han population, the frequency of these two polymorphisms in other ethnic groups must be confirmed and data should be replicated in larger independent cohorts of different ethnicities.

In conclusion, this genetic assessment is the first to evaluate the association of rs10817938 with OSCC risk, prognosis. The C allele at rs10817938 could increase the risk of OSCC and reflect poor prognosis. In addition, the C allele at rs10817938 could decrease XPA production by inhibiting transcriptional activity.

## Supporting Information

S1 Fig Protein relative to GAPDH expression among three OCSS case groups. Circle, OSCC patients with TT; Square, OSCC patients with TC; Triangle, OSCC patients with CC.
(TIF)

S1 Table False-Positive Report Probability Values for association between rs10817938 polymorphism and OSCC risk. (DOC)
