# Supplementary figures and images for: A Functional Polymorphism (rs10817938) in the XPA Promoter Region Is Associated with Poor Prognosis of Oral Squamous Cell Carcinoma in a Chinese Han Population
Source: PLoS One. 2016 Sep 13;11(9):e0160801. doi: 10.1371/journal.pone.0160801 (PMC5021261; doi:10.1371/journal.pone.0160801)

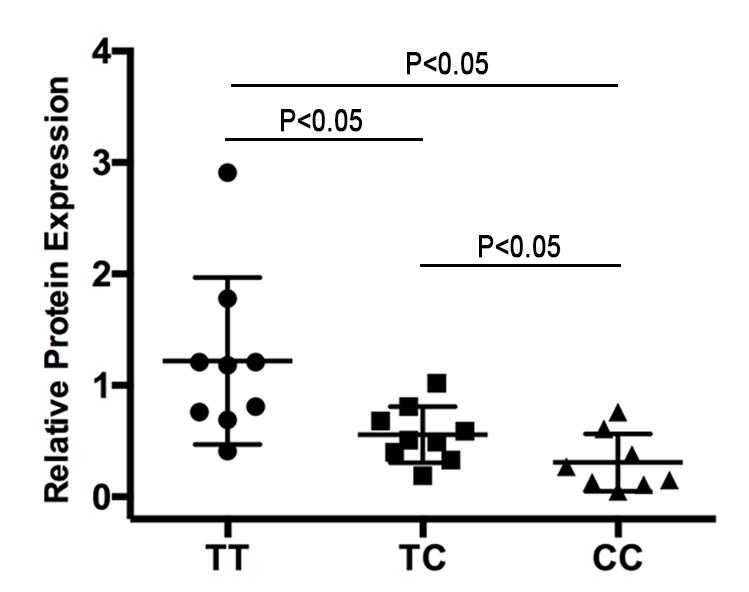

Supplement: S1 Fig — Circle, OSCC patients with TT; Square, OSCC patients with TC; Triangle, OSCC patients with CC. (TIF) [file pone.0160801.s001.tif]
