# Supplementary material for: A Functional Polymorphism (rs10817938) in the XPA Promoter Region Is Associated with Poor Prognosis of Oral Squamous Cell Carcinoma in a Chinese Han Population
Source: PLoS One. 2016 Sep 13;11(9):e0160801. doi: 10.1371/journal.pone.0160801 (PMC5021261; doi:10.1371/journal.pone.0160801)
Supplement: S1 Table — (DOC) [file pone.0160801.s002.doc]

**S1 Table: False-Positive Report Probability Values for association between rs10817938 polymorphism and OSCC risk**

| Genotype/Allele | OR (95%CI) | P value | Statistical Power | Prior Probability | | | | |
| --- | --- | --- | --- | --- | --- | --- | --- | --- |
|  |  |  |  | 0.25 | 0.1 | 0.01 | 0.001 | 0.0001 |
| TT vs TC | 1.42(1.04-1.93) | 0.025 | 0.637 | **0.106** | 0.262 | 0.796 | 0.975 | 0.997 |
| TT vs CC | 2.75(1.32-5.71) | 0.006 | 0.052 | 0.277 | 0.535 | 0.927 | 0.992 | 0.999 |
| TT vs TC+CC | 1.53(1.13-2.06) | 0.005 | 0.448 | **0.033** | **0.092** | 0.528 | 0.919 | 0.991 |
| TT vs CC |  |  |  |  |  |  |  |  |
| Smoking | 3.60(1.20-10.9) | 0.023 | 0.061 | 0.537 | 0.777 | 0.975 | 0.997 | 1.00 |
| TNM Stage | 2.76(1.17-6.51) | 0.020 | 0.082 | 0.428 | 0.692 | 0.961 | 0.996 | 1.00 |
| Lymph Metastasis | 2.78(1.19-6.46) | 0.017 | 0.076 | 0.409 | 0.675 | 0.958 | 0.996 | 1.00 |
| Tumor Differentiation | 4.54(1.94-10.6) | 0.001 | 0.005 | 0.212 | 0.447 | 0.899 | 0.989 | 0.999 |
| T vs C |  |  |  |  |  |  |  |  |
| Smoking | 2.13(1.44-3.15) | 0.001 | 0.040 | **0.011** | **0.034** | 0.276 | 0.794 | 0.975 |
| TNM Stage | 1.54(1.11-2.14) | 0.010 | 0.438 | **0.065** | **0.172** | 0.696 | 0.958 | 0.996 |
| Lymph Metastasis | 1.64(1.18-2.28) | 0.003 | 0.298 | **0.032** | **0.089** | 0.52 | 0.916 | 0.991 |
| Tumor Differentiation | 1.67(1.17-2.37) | 0.004 | 0.274 | **0.043** | **0.118** | 0.596 | 0.937 | 0.993 |
